# Topically applied fullerenols protect against radiation dermatitis by scavenging reactive oxygen species

**DOI:** 10.1186/s11671-023-03869-7

**Published:** 2023-08-15

**Authors:** Hanying Yin, You Gao, Weiguang Chen, Chen Tang, Zihan Zhu, Kun Li, Siyu Xia, Changshun Han, Xiaoyan Ding, Fengkai Ruan, Hanrui Tian, Changfeng Zhu, Suyuan Xie, Zhenghong Zuo, Lixin Liao, Chengyong He

**Affiliations:** 1 State Key Laboratory of Cellular Stress Biology, School of Life Sciences, Faculty of Medicine and Life Sciences, The Plastic and Aesthetic Burn Department, The First Affiliated Hospital, Xiamen University, Xiamen, People’s Republic of China; 2 School of Medicine and School of Biomedical Sciences, Huaqiao University, Xiamen, Fujian China; 3 Xiamen Funano New Materials Technology Co., Ltd., Xiamen, China; 4 State Key Laboratory for Physical Chemistry of Solid Surfaces, Collaborative Innovation Center of Chemistry for Energy Materials, Department of Chemistry, College of Chemistry and Chemical Engineering, Xiamen University, Xiamen, 361005 China

**Keywords:** Fullerenols, Radioprotection, Radiation dermatitis, Antioxidant, Nanomedicine

## Abstract

Adverse skin reactions caused by ionizing radiation are collectively called radiation dermatitis (RD), and the use of nanomedicine is an attractive approach to this condition. Therefore, we designed and large-scale synthesized fullerenols that showed free radical scavenging ability in vitro. Next, we pretreated X-ray-exposed cells with fullerenols. The results showed that pretreatment with fullerenols significantly scavenged intracellular reactive oxygen species (ROS) produced and enhanced the antioxidant capacity, protecting skin cells from X-ray-induced DNA damage and apoptosis. Moreover, we induced RD in mice by applying 30 Gy of X-ray irradiation, followed by treatment with fullerenols. We found that after treatment, the RD scores dropped, and the histological results systematically demonstrated that topically applied fullerenols could reduce radiation-induced skin epidermal thickening, collagen deposition and skin appendage damage and promote hair regeneration after 35 days. Compared with Trolamine cream, a typical RD drug, fullerenols showed superior radiation protection. Overall, the in vitro and in vivo experiments proved that fullerenols agents against RD.

**Supplementary Information:**

The online version contains supplementary material available at 10.1186/s11671-023-03869-7.

## Introduction

Currently, radioisotopes and radiation technology are extensively used in the medical, nuclear energy, industrial, and agricultural fields [1–3]. Since X-rays were discovered by Röntgen in 1895 and first used in the clinic in 1896, radiotherapy has undergone tremendous development [4]. Modern radiotherapy is one of the main modalities of anticancer therapy [5]. External-beam radiation treatments with X-rays, protons, photons, neutron electrons, or carbon ions are utilized in radiotherapy [6, 7].

Although the latest radiation treatments optimize the dose applied to the tumor, the adjacent healthy tissues also suffer from destruction from radiation destruction and inevitable side effects, and these cells may undergo necrosis and apoptosis. The most common side effect of radiotherapy is RD. Up to 95% of cancer patients suffer from RD during radiotherapy [8]. Clinical manifestations of acute RD begin with edematous, warm and typical erythema and then successively appear as dry, as opposed to moist desquamation, and severe skin hemorrhage and necrosis may occur [9]. Histopathological assessment of the irradiated skin shows hyperproliferation of the epidermis and dermis and thickening of the stratum corneum [10]. X-rays can cause DNA damage via direct interactions with DNA and produce excessive ROS or indirect radioactive reactions with water, which further damage basal keratinocytes and hair follicle stem cells [11–13]. Therefore, there is an urgent need to develop highly efficient radioprotective agents for RD to scavenge the excess ROS produced by X-rays.

Currently, numerous existing medications and dressings are used for preventing and treating RD, including triethanolamine, salves or sprays containing superoxide dismutase (SOD), corticosteroids, calendula cream, hyaluronic acid, and sucralfate cream [14]. The use of SOD has some disadvantages, such as poor stability and easy inactivation during long-term storage [15]. Additionally, SOD scavenges only superoxide free radicals (∙O_2_^–^) but cannot eliminate other free radicals, such as hydroxyl radicals (∙OH). Hence, it is essential to develop stable and highly efficient radioprotectants for RD.

Recently, carbon-based nanomaterials, which include fullerenes, graphene, and carbon nanotubes, have shown great promise for the development of new radioprotectants [16]. Fullerenes and polyhydroxylated derivatives such as fullerenols or other derivatives have been utilized in biomedical research. Fullerenols are derivatives of fullerenes that have conjugated double bonds, high electron affinity and polarity, and an excellent ability to scavenge free radicals [17, 18]. Numerous studies have indicated that fullerenols can scavenge ROS and reactive nitrogen species (RNS) due to their structure [19, 20]. Reportedly, fullerenols are potential radioprotective agents against ionizing radiation. The radioprotective abilities of fullerenols were first researched in 2005. The Zhao group indicated that C_60_(OH)_18–22_ had a protective effect on γ-irradiated *Stylonychia. mytilus* at concentrations of 0.06–0.1 mg/mL, and showed that C_60_(OH)_22_, Gd@C_82_(OH)_22_, and C_60_(C(COOH)_2_)_2_ could protect brain microvascular endothelial cells and lung cells against oxidative damage induced by H_2_O_2_ and reduce ROS production [21]. Additionally, Bogdanovic et al. (2008) indicated that pretreatment with 10 μM C_60_(OH)_24_ could protect leukemia cells from 24 Gy of γ-rays and recover SOD and GSH-Px activity [22]. Thus, fullerenols seem to be potential radioprotective agents against ionizing radiation, but there are few studies on the protective effects of fullerenols against RD.

To date, only one study has described the protective effect of fullerenols on skin, which indicated that C_60_(OH)_20_ could effectively block ROS-induced damage and enhance the viability of irradiated human keratinocyte (HaCaT) cells [23]. Sodium hyaluronate hydrogels loaded with C_60_(OH)_20_ were developed for skin administration and were found to effectively alleviate RD by protecting epidermal stem cells [23]. However, the protective effect of fullerenols against RD and its mechanism are still unclear and need to be further studied.

In the present study, we synthesized one hundred grams of fullerenols and investigated their potential radioprotective effects on RD. The fullerenols showed good water solubility and stability and free radical scavenging ability. They reduced the production of intracellular ROS and alleviated the DNA damage and apoptosis of HaCaT and human fibroblast (HFF-1) cells in vitro. Moreover, we found that topical fullerenol application could mitigate RD in mice. Significantly, the radioprotective effects of fullerenols are better than those of Trolamine, a typical RD drug. Overall, we produced highly soluble and stable fullerenols that have excellent radioprotective effects both in vitro and in vivo.

## Materials and methods

### Synthesis of fullerenols

Fullerenols were synthesized under tetrabutylammonium hydride (TBAH)-catalyzed alkaline conditions according to a previously described method with minor modifications [24]. A benzene solution of fullerenes (500 mg in 300 mL) was stirred with 10 mL of 30% aqueous NaOH and 5 mLof 25% TBAH solution in air. Then, 70 mL of 30% hydrogen peroxide was added, and the mixture stirred at 40 °C for 4 h. Within a few minutes, the deep violet benzene solution turned colorless and a brown sludge precipitated out of the solution. The benzene was removed with a separatory funnel and the water phase was retained. The water phase was transferred to a 3000 molecular weight dialysis bag, and the small molecules (NaOH, TBAH and H_2_O_2_) in the water phase were removed by dialysis. Dialysis was continued until the conductivity of the aqueous solutionoutside the dialysis bag was less than 10 μs/cm. The dialysis process may be last for 2–3 days. Furthermore, we removed the aqueous solution from the dialysis bag and evaporated the water. Then, we added 5 mL distilled water and 50 mL ethanol to produce a large amount of a brown solid precipitate, which was filtrated to obtain an earth yellow solid. The solid was washed with ethanol 3 times, and dried to obtain the fullerenols. Under the optimized synthetic conditions, an industrial approach was used to synthesize fullerenols in large quantities (100 g). Six dialysis tanks were used to perform dialysis at the same time, and each could produce 20 g of fullerenol at a time.

### Fullerenol characterization

The samples were diluted with deionized water and cell culture media to 1 mg/mL. The transmission electron microscopy (TEM) images and dynamic light scatting (DLS) and zeta potential measurements of the fullerenols were obtained with a transmission electron microscope (Hitachi HT-7800) and Zetasizer Nano ZS90 from UK Malvern Instruments. Fourier transform infrared (FTIR) spectra of the fullerenols were acquired in I-Br matrices, characterize their molecular structures.

### DPPH and ABTS scavenging activity

The fullerenols were diluted with deionized water to different concentrations (12.5, 25, 100 and 200 mg/L). According to the manufacturer’s protocols, the 1,1-diphenyl-2-picrylhydrazyl (DPPH) radical and 2,2″-azinobis (3-ethyl-benzthiazoline-6-sulfonate) (ABTS) scavenging activities of the fullerenols were measured by DPPH free radical scavenging ability detection kit (BC4755, Solarbio) and ABTS free radical scavenging ability detection kit (BC4770, Solarbio). Different concentrations of fullerenols were mixed with the detection working solutions for DPPH and ABTS at room temperature for 30 min and 6 min respectively. Then the absorbance values at 515 nm (DPPH) and 405 nm (ABTS) were immediately measured by a microplate reader (Tecan, Mannedorf, Switzerland).

The DPPH and ABTS scavenging activities ratio (%) = [A_blank_ − (A_fullerenols_ − A_control)_] ÷ A_blank_ × 100%, where A_blank_ is the absorption value of deionized water without fullerenol and A_control_ is the background absorption value of the fullerenol.

### Cell culture and X-ray irradiation

HaCaT and HFF-1 cells were obtained from the National Institute of Diagnostics and Vaccine Development in Infectious Disease (Xiamen University). Cells were cultured in DME/F-12 medium (Sigma, USA) with 10% fetal bovine serum (FBS) (Gibco, Waltham, MA), 1% nonessential amino acids (NEAAs) (Gibco, Waltham, MA) and 0.1% penicillin and streptomycin (Solarbio, Wuhan, China). HaCaT and HFF-1 cells were seeded into 6-well plates (3 × 10^5^ and 1.5 × 10^5^ cells/well, respectively) and incubated in a humidified incubator under 5% CO_2_ at 37 °C. After 22 h, the cells were carefully washed with phosphate-buffered saline (PBS) and pretreated with FBS-free DME/F-12 medium containing 12.5 or 25 mg/L fullerenols 2 h prior to X-ray irradiation. Then, the cells were irradiated at of 9.445 Gy/min, 160 kV, and 25 mA for different times (165, 330, 660, and 1320 s). X-ray irradiation was performed with an X-ray biological irradiator (RS2000, USA), which delivered total doses of 25, 50, 100 and 200 Gy. After irradiation, the cells were washed with PBS twice and then maintained in fresh FBS-free DME/F-12 medium with the same concentration of fullerenols for another 24 h.

### RD animals and treatment

All procedures involving animals were in compliance with Measures of Xiamen University for the Administration of Experimental Animals. The research protocol was approved by the Ethics Committee of Xiamen University Laboratory Animals Center (XMULAC20170349). BALB/c mice (male, 6–8 weeks old) were obtained from the Beijing Vital River Laboratory Animal Technology and maintained in the Xiamen University Laboratory Animals Center. The left rear leg of each mouse was depilated two days before irradiation. The mice were randomly divided into four groups (n = 18): (1) blank group (untreated); (2) irradiation + vehicle (50% glycerin) group; (3) irradiation + 0.02% fullerenol group; and (4) irradiation + trolamine cream group. Specifically, the left hind leg of each mouse was irradiated with X-rays at a dose of 30 Gy (9.445 Gy/min, 160 kV and 25 mA for 198 s). Two lead shadows were used to shield the other parts of the mice and localize the radiation field (1.2 cm × 1.2 cm).

Fullerenols were dissolved in 50% glycerin at a concentration of 0.02%. Trolamine cream (Laboratoire Medix, France) was used as a positive treatment since it hydrates, drains and cleans irradiated areas well, effectively reducing skin dryness, edema and inflammation, promoting microcirculation, enhancing skin tolerance and accelerating wound healing [25]. Additionally, trolamine can stimulate macrophages and promote collagen synthesis and fibroblast proliferation [26]. After irradiation, the injured skin was smeared twice a day with the designated treatment.

The mice were photographed every day, and nine mice from each group were sacrificed at 20 and 35 days to collect their left hind leg skin for further experiments. The irradiated biopsied skin was fixed in 4% paraformaldehyde, embedded in paraffin wax, and then serially sectioned (thickness, 5 µm). The sections were stained with hematoxylin and eosin (H&E) (Solarbio, Beijing, China) and Masson’s trichrome (Solarbio, Beijing, China). The pathological sections were observed by microscopy (DM4B, Leica, Wetzlar, Germany) at 100 × and 200 × and analyzed by ImageJ. The epidermal and dermal thicknesses were measured at 27 different sites in each section. The collagen deposition areas were measured at 18 different sites in each section.

### Cell viability assay

Cell viability was assessed by 3-(4,5-dimethylthiazol-2-yl)-2,5-diphenyltetrazolium bromide (MTT) assays (Solarbio, Beijing, China). HaCaT and HFF-1 cells were seeded into 96-well plates (1 × 10^4^ and 5 × 10^3^ cells/well, respectively). After 22 h, the cells were treated with fullerenols at 12.5, 25, 50, 100 and 200 mg/L.

Additionally, the radioprotective effect of the fullerenols on HaCaT and HFF-1 cells was detected by MTT assays (Solarbio, Beijing, China). Fullerenols were sonicated for 30 min prior to addition to cell medium. HaCaT and HFF-1 cells were pretreated with FBS-free DME/F-12 medium containing 12.5 and 25 mg/L fullerenols 2 h prior to X-ray irradiation. Then, the cells were irradiated at a dose of 9.445 Gy/min for 330 s. The cells were washed twice with PBS and maintained in fresh FBS-free DME/F-12 medium with the same concentration of fullerenols for 24 h.

After 20 h of treatment, 5 mg/mL MTT was coincubated with 200 μL of FBS-free DME/F-12 medium for 4 h at 37 °C. Then, the medium was removed and the formazan was dissolved in 150 μL of DMSO. After shaking for 10 min, the absorbance values at 490 nm and 580 nm were measured by a microplate reader (Tecan Spark, Switzerland).

### Reactive oxygen species measurements

Intracellular ROS levels were analyzed by DCFH-DA assays (Nanjing Jiancheng Bioengineering Institute, China). HaCaT and HFF-1 cells were seeded in 6-well plates (3 × 10^5^ and 1.5 × 10^5^ cells/well, respectively) and pretreated with 12.5 and 25 mg/L fullerenols for 2 h. Then, the cells were exposed to 50 Gy of X-ray radiation. After incubation for 24 h, the cells were washed with PBS twice and then incubated with 0.5 μM DCFH-DA solution for 30 min at 37 °C. Next, the cells were washed with PBS three times and observed at 100 × magnification by fluorescence microscopy (Olympus IX51, Japan). After exposure to the same conditions, six fields were randomly selected to take photos (n = 3). Finally, the fluorescence intensity (the mean green fluorescence signal intensities) of each picture was semi-quantified by ImageJ software.

### Detection of SOD, CAT, GSH and MDA

Skin cells were pretreated with 12.5 and 25 mg/L fullerenols for 2 h, and then the cells were exposed to 50 Gy of X-rays. After 24 h, the cells were collected, lysed in PBS by ultrasonic pyrolysis and centrifuged at 3500 rpm for 10 min at 4 °C. On the 20th and 35th days after irradiation, the skins of mice with radioactive dermatitis were ground and centrifuged to obtain 2.5% skin homogenates for subsequent experiments.

According to the manufacturer’s protocols (Nanjing Jiancheng Bioengineering Institute, China), the supernatants of the cellular and skin homogenates were mixed with detection working solution for superoxide dismutase (SOD), catalase (CAT) or glutathione (GSH) at 37 °C for a certain length of time and malondialdehyde (MDA) at 95 °C for 90 min. Then the reaction mixtures were centrifuged and transferred to 96-well plates. The absorbance values at 450 nm (SOD), 405 nm (CAT), 405 nm (GSH) and 532 nm (MDA) were measured by a microplate reader (Tecan, Mannedorf, Switzerland).

### Cell apoptosis

HaCaT cells were seeded in 6-well plates and pretreated with 12.5 and 25 mg/L fullerenol for 2 h. Then, the cells were exposed to 50 Gy of X-ray radiation. After 24 h of incubation, the cells were washed with PBS twice and then incubated with 10 μg/mL Hoechst 33,258 solution (Solarbio, Beijing, China) for 30 min at 37 °C. The stained cells were rinsed with PBS three times and assessed by fluorescence microscopy (Olympus IX51, Tokyo, Japan) at 200 × magnification. The stained cells with nuclear fragmentation and condensation were considered apoptotic cells. Finally, the apoptotic cell rate was calculated as follows: apoptotic cell rate (%) = apoptotic cell/total cells × 100%.

#### 8-OHdG assay

To investigate the extent of DNA damage in irradiated HaCaT cells pretreated with fullerenols, the 8-OHdG levels in the cells were assayed by specific enzyme linked immunosorbent assay (ELISA) kit (Chemical Biology Test, Shanghai, China) according to the manufacturers’ instructions.

#### Real-time quantitative reverse transcriptionPCR (qRT–PCR)

RNA was isolated using TRIzol (Servicebio, Wuhan, China), and first-strand cDNA was synthesized by Evo M-MLV RT premix (AG, AG11706). qRT–PCR was performed using a Real-Time PCR System (Bio-Rad, CA, USA). β-Actin was used as a reference gene. The PCR system was operated at 95 °C for 5 min, followed by 40 cycles (95 °C for 15 s; 60 °C for 20 s; 72 °C for 10 s; 80 °C for 15 s), followed by a melt curve analysis. The fluorescence intensity was read at 80 °C. The relative mRNA expression was calculated by the 2^−△△Ct^ methods. *DNA-PK* forward primer: CTAACTCGCCAGTTTATCAATC. *DNA-PK* reverse primer: TTTTTCCAATCAAAGGAGGG. *β-actin* forward primer: CACCAGGGCGTGATGGT. *β-actin* reverse primer: CTCAAACATGATCTGGGTCAT.

#### Statistical analysis

The data are presented as the mean ± standard error (SE). Prism version 7.00 (GraphPad Software, San Diego, California, USA) and SPSS 25.0 were used to perform statistical analyses. Data were analyzed with independent *t* tests. A *p* value of < 0.05 was considered significantly different.

## Results and discussion

### Characterization of fullerenols

Fullerenols, polyhydroxylated derivatives of fullerenes, are formed by introducing hydroxyl groups into fullerenes by hydroxylation reactions [27]. As the number of hydroxyl groups increases, the water solubility of fullerenols increases [28]. We synthesized fullerenols on a large scale and yielded approximately 100 g of each. The fullerenol powder showed great stability and solubility, and their essential properties did not change when stored at room temperature for one year.

**Fig. 1:**
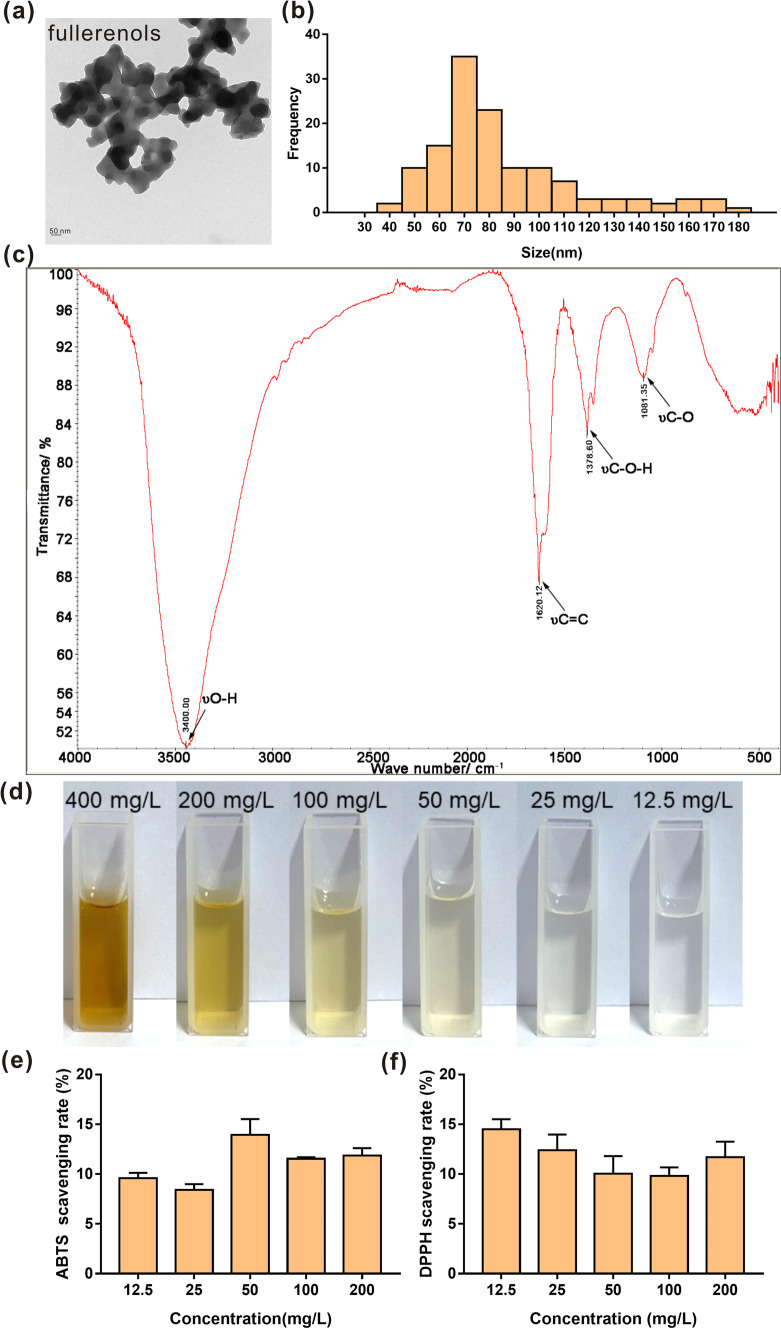
Characterization of fullerenols. **a** TEM images of fullerenols, scale bar = 50 nm. **b** Primary size distribution based on TEM analysis. **c** FTIR spectrum of fullerenols. **d** Typical photos of fullerenols at different concentrations. **e** ABTS radical scavenging capacity. **f** DPPH radical scavenging ability

The fullerenols were characterized by TEM and DLS. The TEM images showed the morphologies of the fullerenols (Fig. 1a) and revealed that the fullerenols are spherical and ~ 85 nm in size (Fig. 1b). The hydrodynamic diameters and zeta potentials of the fullerenols in water and cell culture medium are presented in Table 1. The hydrodynamic diameters in water and DME/F-12 medium were 166.03 ± 9.73 and 704.93 ± 201.52 nm, respectively. The fullerenols had good dispersion in aqueous solutions and cell culture medium (PDI = 0.35 in water and 0.20 in DME/F-12 medium) and a negative surface charge (-8.74 mV in water and − 3.41 mV in DME/F-12 medium). These findings mean that the fullerenols have a stable hydrophilic shell that can maintain a negative surface charge. According to the photographic record of fullerenols (Fig. 1d), it can be also seen that fullerenols have good stability in water.

**Table 1:** The Hydrodynamic sizes and zeta potentials of the fullerenols

	Primary size	Hydrodynamic size (nm)			Zeta potential (mV)	
Sample name		Water	Cell culture media		Water	Cell culture media
Fullerenols	85.18 ± 29.85	166.03 ± 9.73		704.93 ± 201.52	− 8.74 ± 0.73	− 3.41 ± 2.53

The FTIR spectrum of the fullerenols is shown in Fig. 1c. The FTIR spectrum has bands proving the presence of hydroxyl groups attached to the fullerene cage. The spectrum showed that fullerenols have four characteristic infrared absorption peaks, including C−O, C−O−H, and C=C, consistent with the results of Zhao [23]. The FTIR spectrum of the fullerenols demonstrated the distinct characteristic absorption peaks at 1081.35, 1378.6, 1620.12 and 3400 cm^−1^, which corresponded to the stretching of aliphatic C–O bonds, C–O–H stretching, the C=C bond of the aromatic ring and the stretching of the hydroxyl group, respectively.

The above characterization indicated that the fullerenols have conjugated double bonds, which are key to their ability to scavenge free radicals. We evaluated the free radical scavenging abilities of the fullerenols in vitro. DPPH and ABTS are representative model radicals used to evaluate the ability to scavenge free radicals [29]. When the fullerenol concentrations were 12.5 and 25 mg/L, the ABTS and DPPH scavenging rates were 9.59% and 14.84% and 8.40% and 12.40%, respectively (Fig. 1e, f). These data show that the fullerenols have the ability to scavenge DPPH and ABTS in vitro. Our results are similar to those of Hao, who indicated that the scavenging effect of DPPH radicals by fullerenols is below 29% when the fullerenol concentration is below 200 μg/mL [30]. Additionally, the DPPH radical scavenging effect is below 25% when the fullerenol concentration is below 50 μg/mL [23]. While, the hydroxyl radicals scavenging rate is below 6% when the fullerenol concentration is below 50 mg/L (Additional file 1: Fig. S2). In need, our synthetic fullerenols have low scavenging effect on hydroxyl radicals. The scavenging effects of hydroxy radicals by fullerenols might be related to synthetic method and the distribution of hydroxyls. Wang et al. found that the ·OH^−^ scavenging activity depends on the distribution of hydroxyls according to the calculations for ten C_60_(OH)_24_ isomers [31]. We used an industrial approach to synthesize highly soluble and stable fullerenols in large quantities (> 100 g) under the optimized synthetic conditions. Different synthetic method might influence the distribution of hydroxyls. Our synthetic fullerenols show low scavenging effect on hydroxyl radicals, but they might have O_2_^•−^, ^1^O_2,_ and H_2_O_2_^−^ scavenging abilities.

### Radioprotective effects of fullerenols on skin cells

**Fig. 2:**
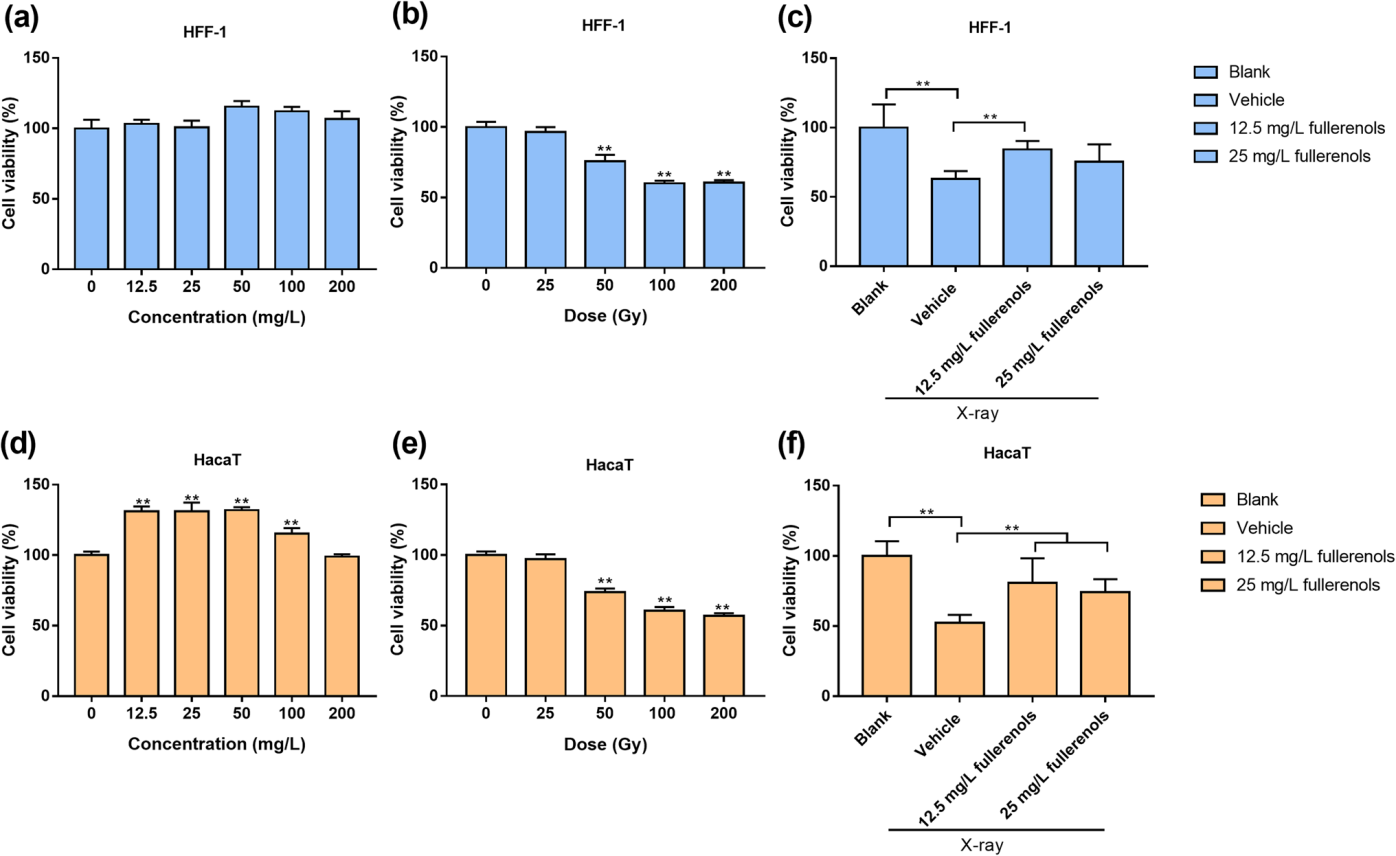
The radioprotective effects of the fullerenols on X-ray exposed HaCaT and HFF-1 cells. Viability of HFF-1 (**a**) and HaCaT cells (**d**) treated with different concentrations of fullerenols for 24 h. Viability of HFF-1 (**b**) and HaCaT cells (**e**) treated with different doses of X-rays (0, 25, 50, 100, 200 Gy) for 24 h. Viability of HFF-1 (**c**) and HaCaT cells (**f**) with or without fullerenols after 24 h irradiation (50 Gy). Pretreatment with 12.5 and 25 mg/L fullerenols lasted for 2 h. * *p* < 0.05, ** *p* < 0.01

To study the radioprotective effects of fullerenols, we first examined the safe concentration of fullerenols in HFF-1 and HaCaT cells. After the cells were treated with varying concentrations of fullerenols (12.5, 25, 50, 100 and 200 mg/L), it was demonstrated that they have no or scarce toxicity to HFF-1 and HaCaT cells at concentrations below 200 mg/L (Fig. 2a, d).

As shown in Fig. 2b, e, X-ray irradiation dose-dependently decreased the cell viability of HFF-1 and HaCaT cells. X-ray irradiation at 50 and 100 Gy significantly decreased the viabilities of HFF-1 and HaCaT cells by 0.24 and 0.25-fold and 0.40-, 0.39-fold, respectively, compared to the blank group (no irradiation). Based on the cell viability results, we chose 50 Gy as the moderate dose for further experiments.

As shown in Fig. 2c, f, treatment with 12.5 and 25 mg/L fullerenols for 2 h prior to X-ray irradiation significantly increased the viability of HFF-1 and HaCaT cells by 0.34- and 0.54-fold, respectively, compared to the vehicle group (irradiated only). These results showed that the fullerenols could effectively protect HFF-1 and HaCaT cells from irradiation.

### Fullerenols mitigated X-ray-induced oxidative stress in skin cells

**Fig. 3:**
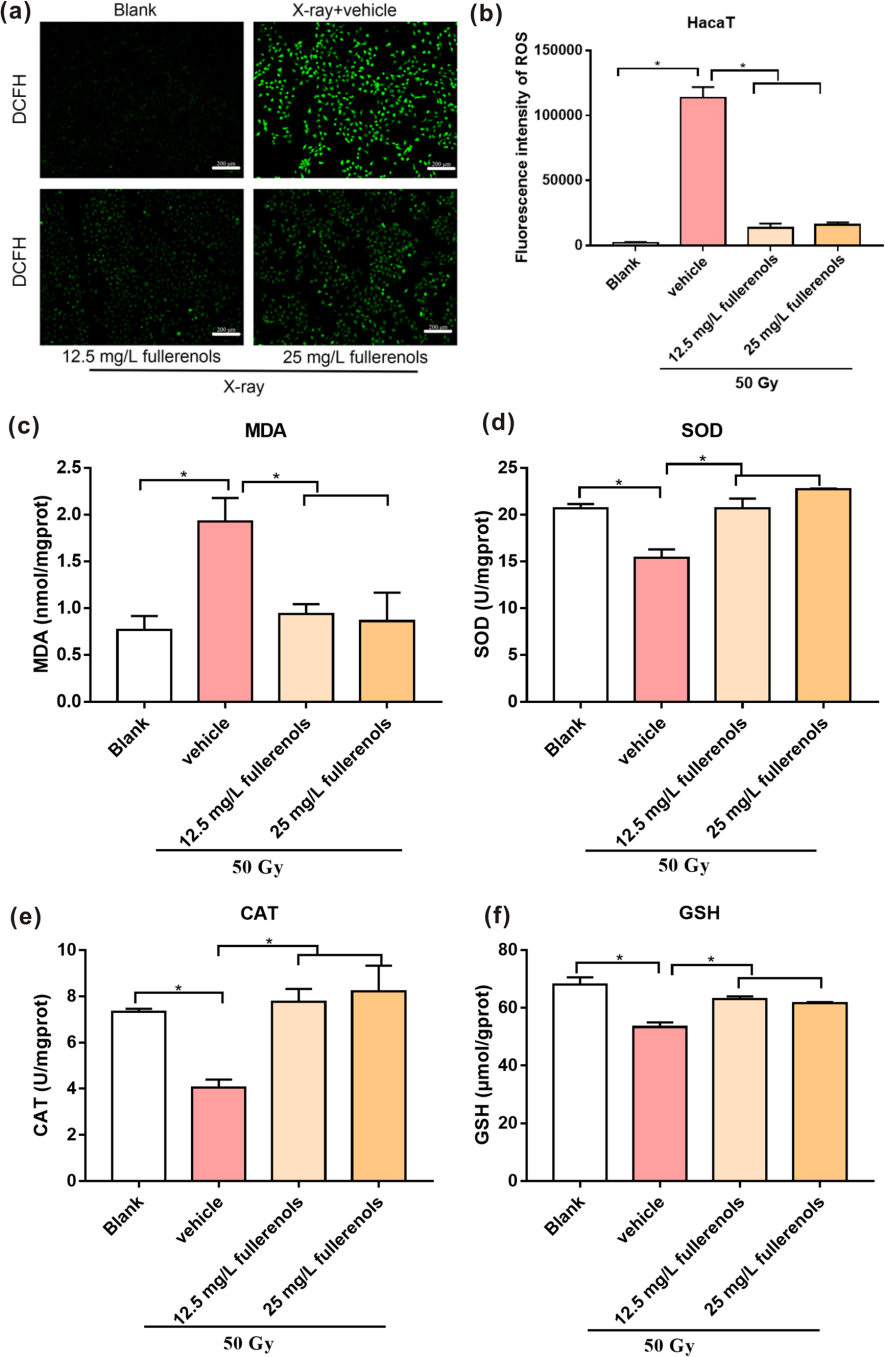
Fullerenols mitigated oxidative stress damage in HaCaT cells 24 h after exposure to 50 Gy of X-ray irradiation and pretreatment with 12.5 and 25 mg/L fullerenols for 2 h. **a** Fluorescence images of HaCaT cells stained with 0.5 μM DCFH-DA 24 h after irradiation. **b** The fluorescence intensity of the HaCaT cells was quantified by ImageJ software (mean ± SE, n = 3). **c** MDA levels (n = 4). **d** SOD activity (n = 3). **e** CAT activity (n = 3). **f** GSH content (n = 4). * *p* < 0.05, ** *p* < 0.01

Encouraged by the above radioprotective efficacy of fullerenols in HFF-1 and HaCaT cells, the potential mechanisms of the radioprotection were further explored. Twenty-four hours after irradiation, ROS production was detected. As shown in Fig. 3, 50 Gy of X-ray irradiation resulted in an evident elevation in ROS production in HaCaT cells compared with the blank group, as the X-ray-induced ROS levels were 45.51-fold higher (Fig. 3a, b). Treatment with 12.5 and 25 mg/L fullerenols significantly inhibited the increase in the ROS level. This finding further showed that fullerenols could suppress ROS production caused by X-ray irradiation.

MDA is a product of lipid peroxidation and can be used as a biomarker of oxidation. As shown in Fig. 3c, 50 Gy of X-ray irradiation caused a significant increase in MDA levels in HaCaT cells compared with the blank treatment. In addition, SOD and CAT are antioxidant enzymes and GSH is an endogenous antioxidant molecule. Evident attenuation of SOD activity, CAT activity and GSH content in HaCaT cells was caused by 50 Gy of X-ray irradiation compared with the blank group (Fig. 3d, e, f). However, fullerenols could overcome or suppress theses effects. As shown in Fig. 3c, 12.5 and 25 mg/L fullerenols significantly decreased the X-ray-induced MDA level by 0.49- and 0.46-fold, respectively, compared to the vehicle group. Furthermore, fullerenols at the same concentration significantly increased the SOD activity, CAT activity and GSH content in HaCaT cells compared with the vehicle group (Fig. 3d, e, f). Collectively, fullerenols had the capacity to reduce the generation of X-ray-induced oxidative products and enhance antioxidative activity.

### Fullerenols inhibit X-ray induced DNA damage in HaCaT cells

**Fig. 4:**
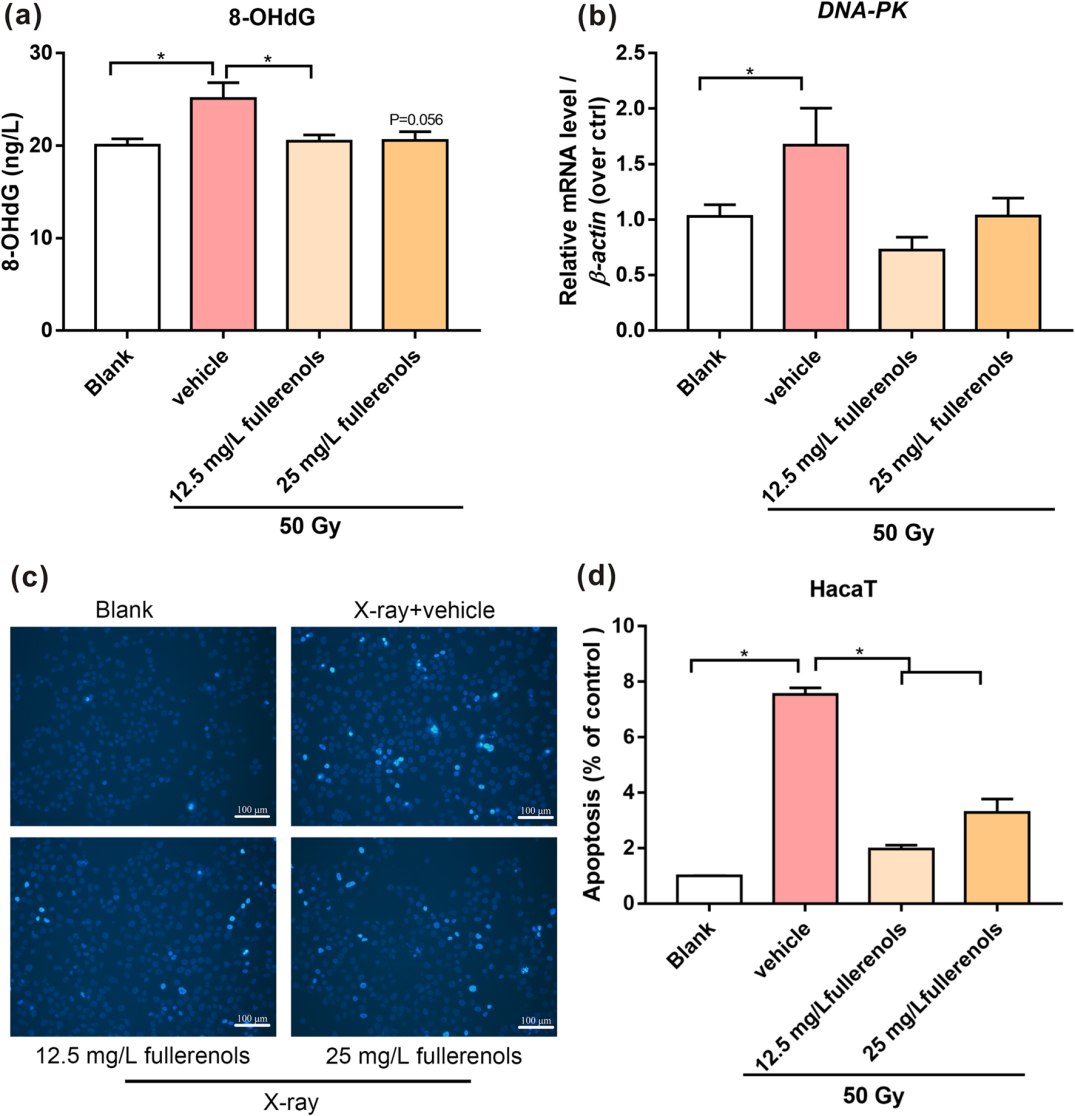
Fullerenols inhibited X-ray-induced HaCaT cell DNA damage and apoptosis. **a** The 8-OHdG level in HaCaT cells 24 h after irradiation (n = 4). **b** The mRNA expression of *DNA-PK* (n = 4). **c** Fluorescence images of HaCaT cells stained with 10 µg/mL Hoechst 33258 solution 24 h after irradiation. Scale bar, 100 μm. **d** Quantitative analysis of apoptotic cells (n = 3). * *p* < 0.05, ** *p* < 0.01

HaCaT cells were pretreated with 12.5 and 25 mg/L fullerenols 2 h prior to irradiation and then collected to detect the 8-OHdG content 24 h after irradiation. 8-OHdG is a product of DNA oxidative damage. It was previously shown that X-ray irradiation increased the 8-OHdG level in DNA in a cells, animals, and humans [32, 33]. As shown in Fig. 4a, 50 Gy of X-ray irradiation significantly increased the 8-OHdG content compared with the blank group. However, treatment with 12.5 mg/L fullerenols markedly decreased the 8-OHdG content compared with the vehicle group. These results indicated that fullerenols could prevent cell DNA damage by effectively scavenging ROS.

Cells exposed to X-rays can experience DNA damage, including DNA double-strand breaks (DSBs), single-strand breaks (SSBs) and modified bases [34]. It is known that DNA damage can activate extensive signaling molecules and influence the transcription of many genes, such as DNA-dependent protein kinase (DNA-PK) [35, 36]. In response to ionizing radiation-induced DNA injury, DNA-PKcs can activate p53 to induce apoptosis in fibroblasts and mice [37, 38].

Moreover, the expression levels of DNA repair marker genes were measured by qRT–PCR in HaCaT cells 24 h after irradiation (Fig. 4b). Irradiation with 50 Gy of X-ray caused a conspicuous increase in *DNA-PK* mRNA expression compared with that in the blank group. However, the mRNA expression of *DNA-PK* in the 12.5 and 25 mg/L fullerenol groups was similar to that in the blank group. These results demonstrated that fullerenols could protect against X-ray irradiation-induced DNA damage by scavenging the ROS produced after irradiation.

### Fullerenols inhibit X-ray induced HaCaT cell apoptosis

X-rays produce excess ROS, damage DNA, inhibit cell proliferation, and eventually lead to cell apoptosis [39]. HaCaT cells were pretreated with 12.5 and 25 mg/L fullerenols under the optimal experimental conditions, and then the cells were stained with Hoechst 33258 24 h after X-ray irradiation. As shown in Fig. 4c, the stained cells with nuclear fragmentation and condensation were considered apoptotic cells. X-ray irradiation markedly increased the percentage of apoptotic cells, but an obvious attenuation of apoptotic cells resulted after treatment with fullerenols (Fig. 4d). These results suggested that fullerenols showed radioprotective effects on skin cells by reducing apoptosis by scavenging ROS.

It has been demonstrated that X-rays can trigger DNA damage and cellular oxidative stress, leading to the excess production of ROS [40]. Cell damage caused by X-rays is mainly due to highly destructive free radicals generated by radiolysis of the cellular aqueous milieu [41]. Free radicals are converted into ROS in an aerobic environment and are responsible for indirect radiation damage. ROS have a profound impact on the pathological and physiological functions of cells, tissues and even entire organisms [42, 43]. The excess ROS produced by X-ray irradiation induces adverse damage to proteins, DNA, RNA and lipids, resulting in DNA damage, mitochondrial dysfunction, the production of lipid peroxides and even cell death [44]. Zhang et al. demonstrated that 2 and 5 Gy of X-ray irradiation induced high levels of intracellular ROS in HaCaT cells 2, 4 and 12 h after irradiation [45]. Irradiation also caused excess intracellular ROS production in HaCaT cells and activated cleaved Caspase3, eventually leading to apoptosis [13]. Qian et al. proved that 8 Gy of X-ray irradiation significantly increased ROS in HaCaT cells, eventually leading to apoptosis [46]. Lagadu et al. found that 6 Gy of irradiation significantly increased the 8-OHdG levels in HFF-1 cells, leading to DNA damage [47]. Consistent with these studies, our results demonstrated that 50 Gy of X-ray irradiation resulted in an obvious increase in ROS production in HaCaT cells in addition to an increased MDA content and attenuation of SOD activity, CAT activity and GSH content, followed by DNA damage and apoptosis. Collectively, we found that pretreatment with fullerenols lead to significant scavenging of intracellular ROS and an enhancement in antioxidant capacity, thus protecting skin cells from X-ray-induced DNA damage and apoptosis.

### Fullerenols promote healing in RD mice

**Fig. 5:**
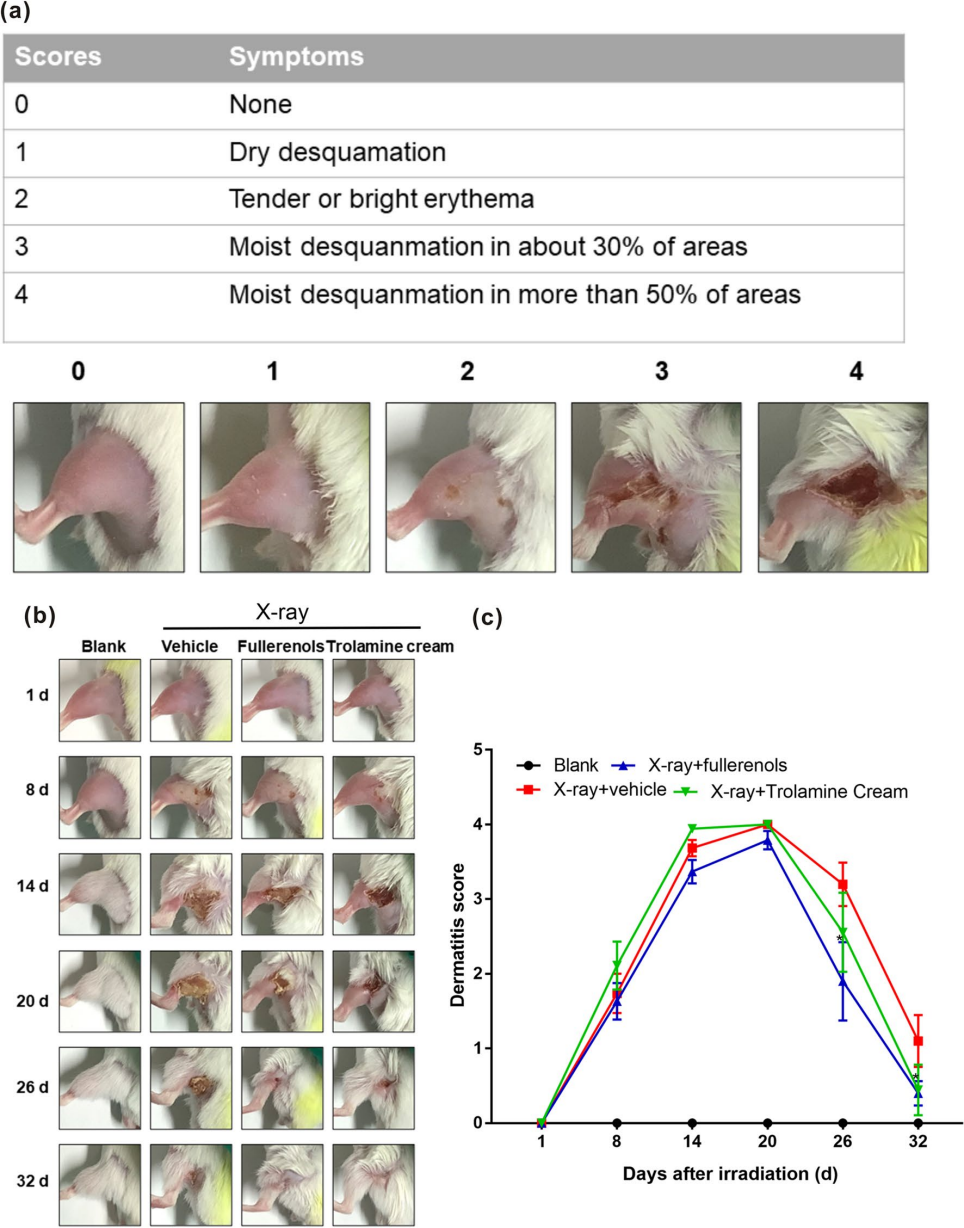
The effects of fullerenols on RD mice. **a** Representative photographs of the left hind leg skin representing the Radiation Therapy Oncology Group (RTOG) scores. **b** Representative photographs taken of the left hind leg skins of RD BALB/c mice after different treatments. **c** RTOG scores of RD mice on different days after the indicated treatments. n ≥ 9, * *p* < 0.05

Inspired by the satisfying radioprotective performance of the fullerenols on skin cells that scavenge the excess ROS resulting from irradiation, we then evaluated the fullerenols for skin radioprotection in vivo by visually observing the irradiated skin area changes on the left hind legs of BALB/c mice and their corresponding histopathological phenomena. Mice were irradiated on the left hind leg with 30 Gy, and we estimated the effects of topical fullerenol application on RD. Acute RD was scored according to the Radiation Therapy Oncology Group (RTOG) criteria (Fig. 5a) [48, 49].

As shown in Fig. 5b, c, the skin in the irradiation field exhibited moist desquamation 5 to 8 days after irradiation. In all groups, the skin gradually became completely desquamated and ulcerated from 14 to 20 days after irradiation. By the 20th day, the largest ulcer area with serious injury occurred in the X-ray group, and the ulcer area on the skin in the trolamine cream + irradiation group was larger than that in the fullerenol + irradiation group. On the 21st and 35th days after irradiation, the ulcerated skin was finally healed by scar formation. Fullerenol and trolamine cream treatment promoted faster healing in RD mice. Similarly, the scores in the 0.02% fullerenol treatment group were significantly lower than those in the irradiation group on the 26th and 32nd days after irradiation. Trolamine cream showed a similar trend to 0.02% fullerenol treatment on the 21st day after irradiation. These results demonstrated that topical fullerenol application could promote healing in RD mice.

**Fig. 6:**
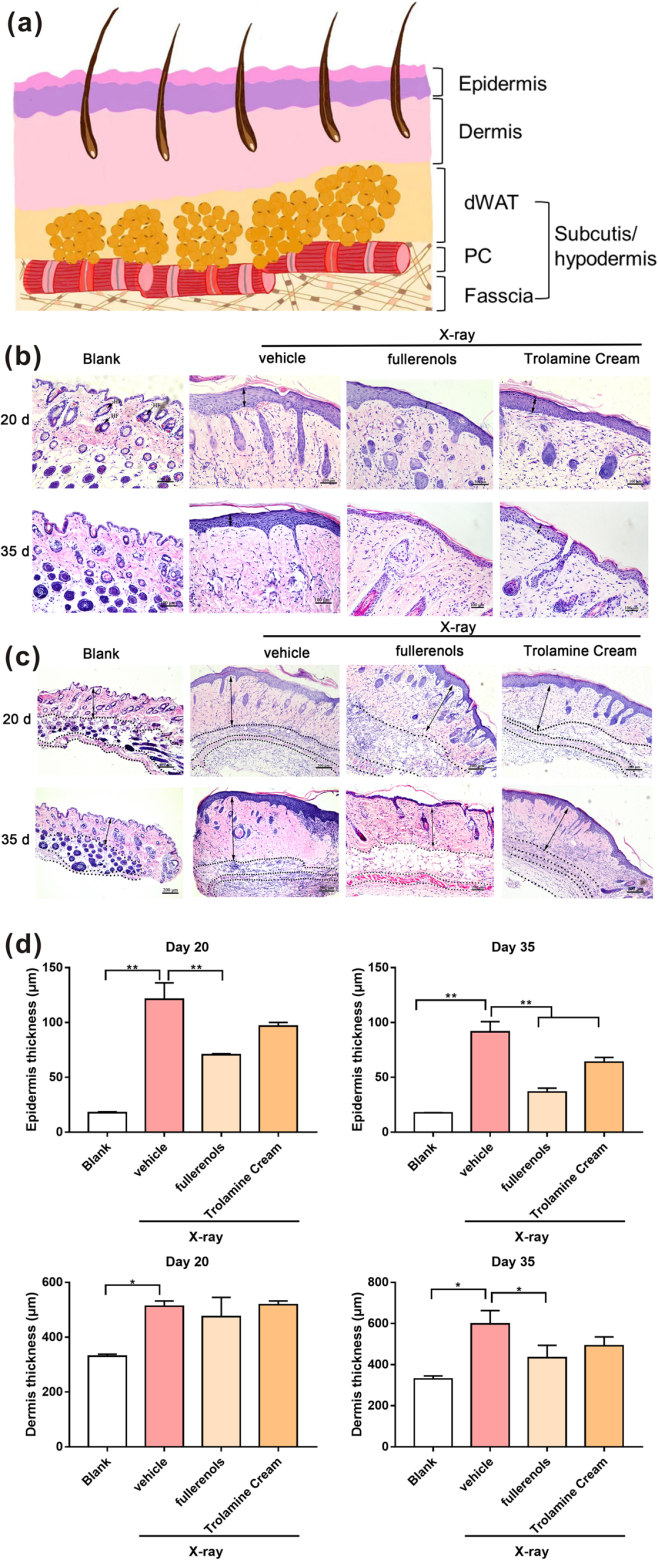
Fullerenols reduced the radiation-induced thickening of the epidermis and dermis in RD mice. **a** Schematic of the skin structure. dWAT, dermal white adipose tissue; PC, panniculus carnosus muscle. Representative H&E staining images of the skin epidermis (**b**) and dermis (**c**) in skin tissues after the indicated treatments. **d** Measurement of the epidermal and dermal thicknesses in the skin tissues collected 20 and 35 days after irradiation. *p < 0.05, **p < 0.01

As displayed in Fig. 6, H&E staining showed that both the epidermis and dermis were thicker in the irradiated skin tissues than in the unirradiated tissues on the 20th and 35th days. Quantification of the data indicated that 20 days after 30 Gy of X-ray irradiation there was a marked increase in the epidermal (from 17.49 ± 0.44 to 121.16 ± 8.70 µm) and dermal (from 325.29 ± 6.42 to 512.68 ± 11.31 µm) thicknesses, whereas topical fullerenol application mitigated the irradiation-induced epidermal thickening (Fig. 6b, c, d). After treatment with 0.02% fullerenol, the epidermal thickness was reduced to 70.58 ± 0.99 µm (Fig. 6d), and the epidermal thickness was reduced to 96.72 ± 3.34 µm with trolamine cream treatment. On the 35th day, after treatment with 0.02% fullerenol, the epidermal thickness was significantly reduced to 36.73 ± 3.42 µm (Fig. 6d). In addition, the thickness of the dermis was prominently reduced to 433.71 ± 60.08 µm compared with that in the irradiation + vehicle (50% glycerin) group. The epidermal thickness was dramatically reduced to 63.81 ± 4.32 µm after trolamine cream treatments (Fig. 6d). Collectively, topical fullerenol application could reduce epidermal thickening caused by irradiation.

**Fig. 7:**
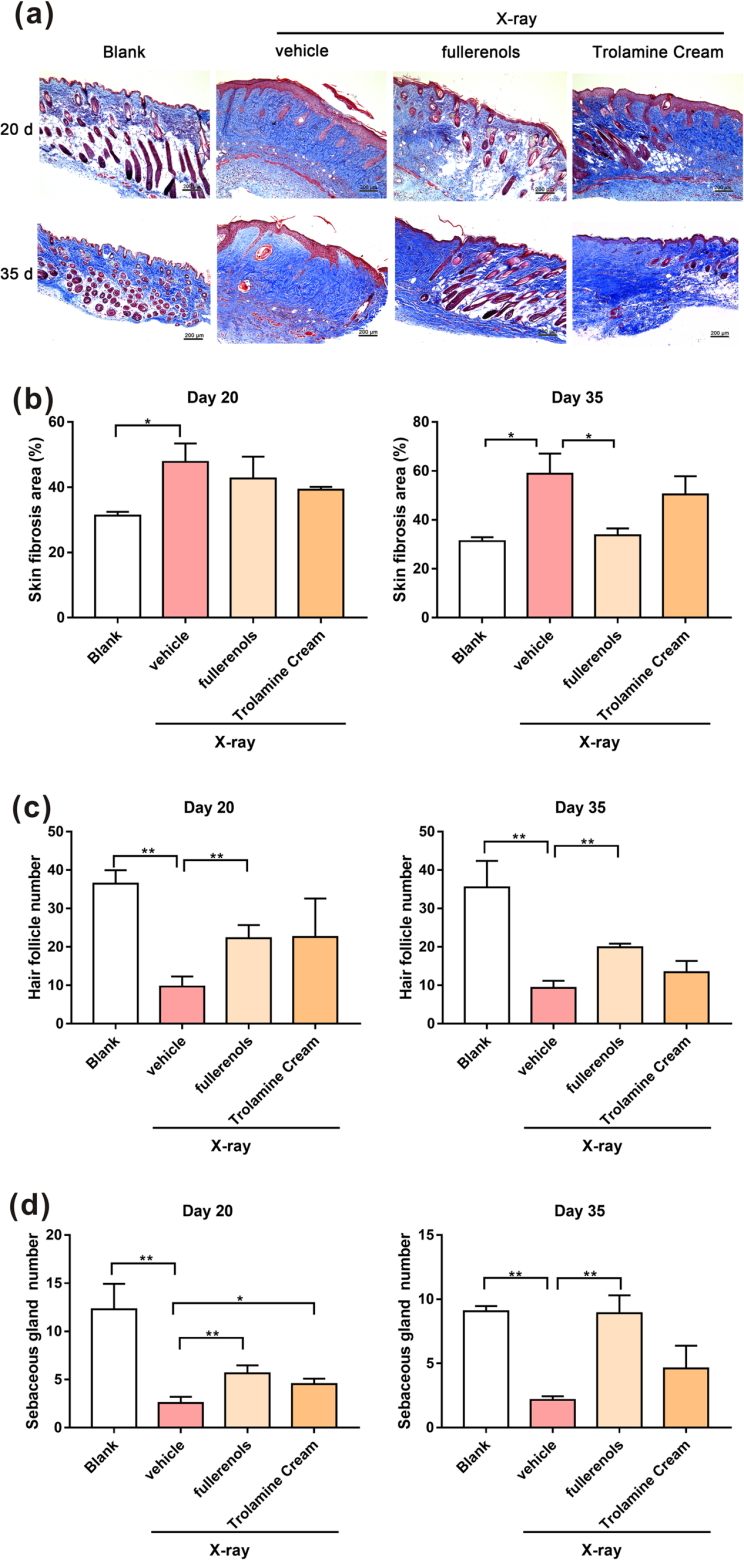
Fullerenols alleviated radiation-induced collagen deposition and skin appendage damage. **a** Representative Masson’s trichrome staining photos of skin after different treatments. **b** Statistical analysis of the Masson staining results. Statistical analysis of the number of hair follicles (**c**) and sebaceous glands (**d**) 20 and 35 days after irradiation (n = 3). Scale bar, 200 μm. * *p* < 0.05, ** *p* < 0.01

As displayed in Fig. 7, Masson staining showed that the irradiated skin tissues exhibited collagen deposition on the 20th and 35th days after irradiation. Quantification of the data showed that on the 20th and 35th days after X-ray irradiation there were marked increases in collagen deposition (0.35- and 0.47-fold, respectively). On the 20th day, topical fullerenol and tryptamine cream application alleviated collagen deposition, with no significant differences. On the 35th day, topical fullerenol application significantly reduced collagen deposition (Fig. 7b). Moreover, the sebaceous glands and hair follicles were damaged by X-ray irradiation; the number of hair follicles and sebaceous glands decreased, their morphology changed and they atrophied (Fig. 7a, c, d). Interestingly, except for the X-ray group, hair regeneration to different degrees was observed in all groups. The fastest hair regeneration was observed in the fullerenol + irradiation group. Topical fullerenol application significantly protected the hair follicles and sebaceous glands (Fig. 7c, d). By the 35th day, trolamine cream treatment significantly protected the sebaceous glands. These results further confirmed that fullerenols alleviate radiation-induced skin collagen deposition and skin appendage damage and promote hair regeneration.

### Fullerenols mitigated oxidative stress in RD mouse skin

As described above, we found that fullerenols have high radioprotective efficacy against radiation-induced skin injury in vivo. Acute RD is caused by direct tissue damage from radiation accompanied by the progression of the inflammatory response with a simultaneously occurring recovery process [50]. Skin injury caused by X-rays is mainly related to oxidative stress. X-rays produce excess ROS and antioxidant enzymes such as superoxide dismutase, glutathione peroxidase, and thioredoxin, which protect the skin cells from radiation-induced oxidative stress [51]. We explored the effects of fullerenols on oxidative stress in RD mouse skin.

**Fig. 8:**
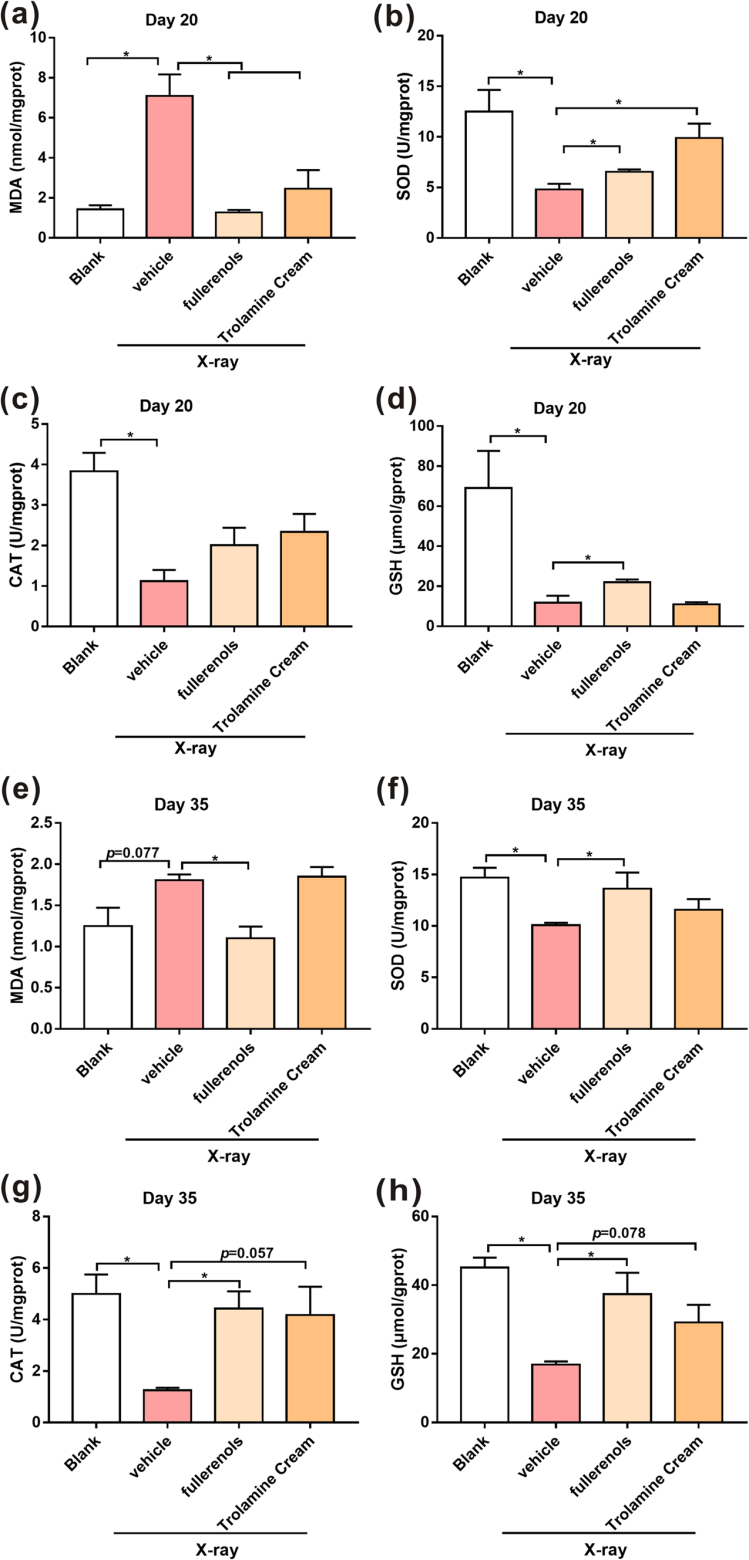
Fullerenols mitigated oxidative stress damage in the skin of RD mice on the 20th and 35th days after irradiation. Twentieth day **a**–**d** after irradiation. **a** MDA levels. **b** SOD activity. **c** CAT activity. **d** GSH contents. Thirty-fifth day (E–H) after irradiation. **e** MDA levels. **f** SOD activity. **g** CAT activity. **h** GSH contents. n = 3, * *p* < 0.05

As shown in Fig. 8a, e, on the 20th and 35th days after irradiation, there were significant increases in MDA levels in RD mouse skin compared with the blank group (3.95- and 0.45-fold, respectively). In addition, there was an evident attenuation of SOD activity, CAT activity and GSH content in RD mouse skin compared with the blank group. On the 20th and 35th days, fullerenols significantly decreased the X-ray-induced MDA level by 0.82- and 0.39-fold, respectively, compared to the vehicle group (irradiated only and without fullerenol treatment). Furthermore, on the 20th and 35th days, fullerenols at the same concentration significantly increased the SOD activity, CAT activity and GSH content by 0.36- and 0.35-fold, 0.79- and 3.52-fold, and 0.87- and 1.21-fold, respectively (Fig. 8b–h). Collectively, topical fullerenol application reduced skin oxidative stress caused by irradiation and enhanced skin antioxidative activity, thus reducing RD.

In this study, a typical RD treatment, trolamine cream, was used as the positive control. Compared with trolamine cream, fullerenols exhibited superior radioprotective effects. Topical fullerenol application promoted faster healing in RD mice, was more effective in reducing epidermal thickening, collagen deposition and skin oxidative stress, and provided more significant protection to the hair follicles and sebaceous glands. Fullerenols and trolamine cream likely utilize different mechanisms. Fullerenols exert radioprotective effects on skin by scavenging X-ray-induced ROS, reducing oxidative stress, and inhibiting skin cell apoptosis and DNA damage. Trolamine cream likely effectively reduces inflammation, stimulates macrophages and promotes collagen synthesis and fibroblasts, thus accelerating wound healing in RD. Fullerenols and trolamine cream both showed radioprotective effects against RD. The anti-inflammatory effects of fullerenols are worth further exploration.

To date, molecular drugs for the prevention and treatment of RD have the drawbacks of short systemic circulation time in vivo, fast metabolism and low bioavailability, which lead to low efficacy, and it is difficult to achieve a satisfactory protective effect under the toxicity threshold [52]. Numerous in vitro and in vivo experiments have shown that topical SOD application can effectively alleviate acute RD and treat skin fibrosis with few side effects but with a short half-life and high cost [53–55]. However, nanomaterials have many superior properties inducing good stability under various physiological conditions, a relatively long systemic circulation time in vivo, a low clearance rate and effective absorption by cells [56]. Fullerenols showed good stability and free radical scavenging abilities. In addition, our results indicated that fullerenols showed powerful radioprotective effects against RD. Zhao et al. demonstrated that fullerenols have good biosafety for skin and low systemic toxicity in mice [23]. The use of fullerenols is highly feasible for preventing and treating RD in the clinic.

## Conclusions

In summary, we synthesized fullerenols on a one hundred gram-scale that showed great stability, water solubility and antioxidant properties. Our study systematically demonstrated that fullerenols showed radioprotective effects on skin by scavenging the excess ROS induced by irradiation. In vitro experiments indicated that pretreatment with fullerenols could effectively scavenge X-ray-induced ROS and inhibit skin cell apoptosis and DNA damage. The results of in vivo experiments demonstrated that topical fullerenol application could alleviate RD and promote healing of radiation dermatitis mice by reducing skin oxidative stress caused by X-rays and enhancing skin antioxidant activity. Topical fullerenol application could reduce radiation-induced skin epidermal thickening, collagen deposition and skin appendage damage and promote hair regeneration. Compared with trolamine cream, a typical RD drug, fullerenols showed superior radiation protection. Thus, fullerenols could be ideal radioprotective agents against high-dose X-rays with low cytotoxicity. In the future, fullerenols could be valuable for use during radiotherapy to prevent RD.

### Supplementary Information

**Additional file 1.**

## Data Availability

The authors declare that the data supporting the findings of this study are available within the paper. Should any raw data files be needed in another format they are available from the corresponding author upon reasonable request.
