# Supplementary material for: Topically applied fullerenols protect against radiation dermatitis by scavenging reactive oxygen species
Source: Discov Nano. 2023 Aug 15;18(1):101. doi: 10.1186/s11671-023-03869-7 (PMC10427596; doi:10.1186/s11671-023-03869-7)
Supplement: Supplementary file 1 — Additional file 1. [file 11671_2023_3869_MOESM1_ESM.docx]

**Supporting Information**

**Topically applied fullerenols protect against radiation dermatitis by scavenging reactive oxygen species**

Hanying Yin ^a,1^, You Gao ^a,1^, Weiguang Chen ^b,1^, Chen Tang ^a^, Zihan Zhu ^a^, Kun Li ^a^, Siyu Xia ^a^, Changshun Han ^a^, Xiaoyan Ding ^a^, Fengkai Ruan ^a^, Hanrui Tian ^d^, Changfeng Zhu ^c^, Suyuan Xie ^d^, Zhenghong Zuo ^a^, Lixin Liao ^a,*^, Chengyong He ^a,*^

^a^ State Key Laboratory of Cellular Stress Biology, School of Life Sciences, Faculty of Medicine and Life Sciences, The Plastic and Aesthetic Burn Department, The First Affiliated Hospital, Xiamen University, Xiamen, PR China

^b^School of Medicine and School of Biomedical Sciences, Huaqiao University, Xiamen, Fujian, China.

^c^ Xiamen Funano New Materials Technology Co., Ltd., Xiamen, China.

^d^ State Key Laboratory for Physical Chemistry of Solid Surfaces, Collaborative Innovation Center of Chemistry for Energy Materials, Department of Chemistry, College of Chemistry and Chemical Engineering, Xiamen University, Xiamen 361005, China.

*Phone: +86 15860784275 E-mail: [hecy@xmu.edu.cn](mailto:hecy@xmu.edu.cn) **&** [liaolixn@126.com](mailto:liaolixn@126.com)

^1^ These authors contributed to this work equally.

- 1. Thermal Gravimetric Analysis (TGA) and ICP of fullerenols

Thermogravimetric analysis (TGA) was used to evaluate the thermal stability of C_60_(OH)_x_ for the estimation of –OH functionalities attached to the C_60_ cage and also to evaluate the stability of the fullerenols.

As shown in Figure S1, two distinct weight-loss stages were observed. Where loss of hydroxyl addends occurs from 150 to 570 °C and the degradation of the C_60_ cage begins at >570 °C.

We quantitatively detected the content of C, H, N, O elements through elemental analysis and Na content through ICP, and inferred the molecular formula of the synthesized compound through TGA. The mass fractions of corresponding elements C, H, O, Na are 43.13%, 3.06%, 42.79% and 7.8%, and the content of water is 9.05%. The next step is to calculate the number of corresponding atoms as 60, 51.1, 44.6, 5.7, and 8.4, respectively. Finally, we calculate the average molecule of the compound and inferred the molecular as C_60_H_34.4_O_36.2_Na_5.7_·8.4H_2_O. As shown in Table S2.

Table S1. The Hydrodynamic size of fullerenols

|  | **water** | | **Cell culture media** | |
| --- | --- | --- | --- | --- |
|  | The Hydrodynamic size (nm) | PDI | The Hydrodynamic size (nm) | PDI |
| Test 1 | 154.9 | 0.399 | 517.1 | 0.197 |
| Test 2 | 170.3 | 0.334 | 679.9 | 0.245 |
| Test 3 | 172.9 | 0.302 | 917.8 | 0.164 |
| Mean±SE | 166.03±9.73 | 0.35±0.05 | 704.93±201.52 | 0.20±0.04 |

Table S2. The elemental analysis of fullerenols

|  | C | H | N | O | H_2_O | Na |
| --- | --- | --- | --- | --- | --- | --- |
| Methods | elemental analysis | | | elemental analysis | TGA | ICP |
| mass fraction % | 43.13 | 3.06 | NA | 42.79 | 9.05 | 7.8 |
| relative atomic (molecular) mass | 12 | 1 |  | 16 | 18 | 23 |
| Number of atoms | 60 | 51.1 |  | 44.6 | 8.4 | 5.7 |
| average molecular formula |  | C_60_H_34.4_O_36.2_Na_5.7_·8.4H_2_O | | | |  |


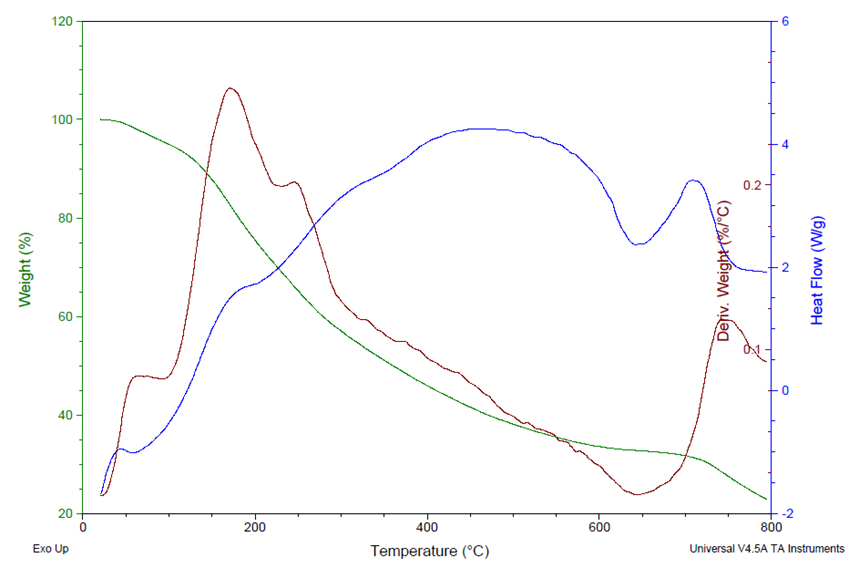


**Fig. S1** The thermogravimetric analysis (TGA) of fullerenols


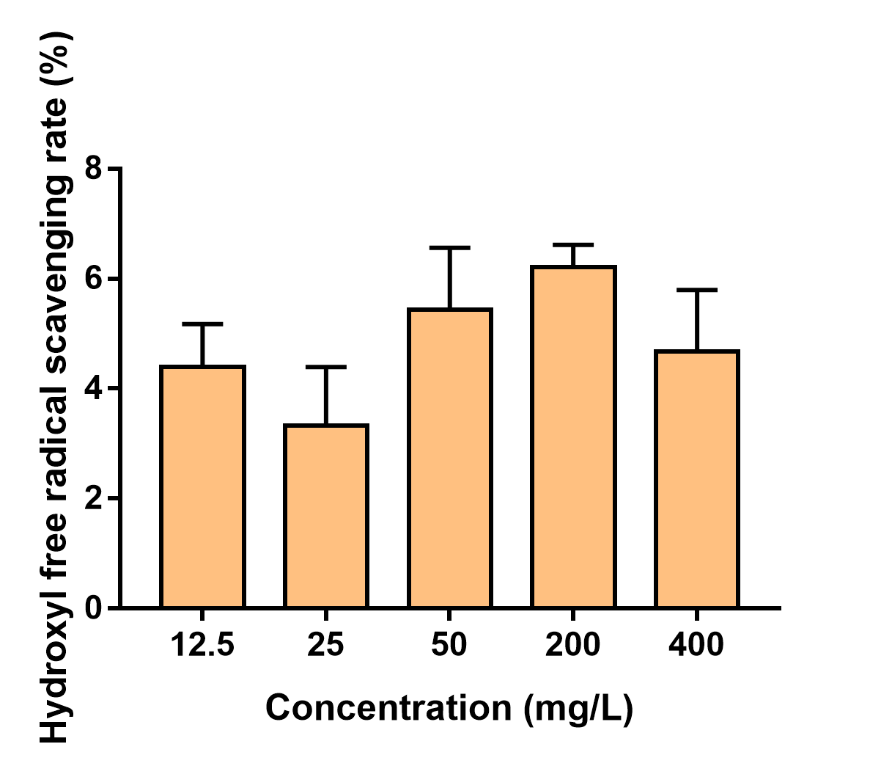


**Fig. S2** Hydroxyl free radical scavenging rate of fullerenols


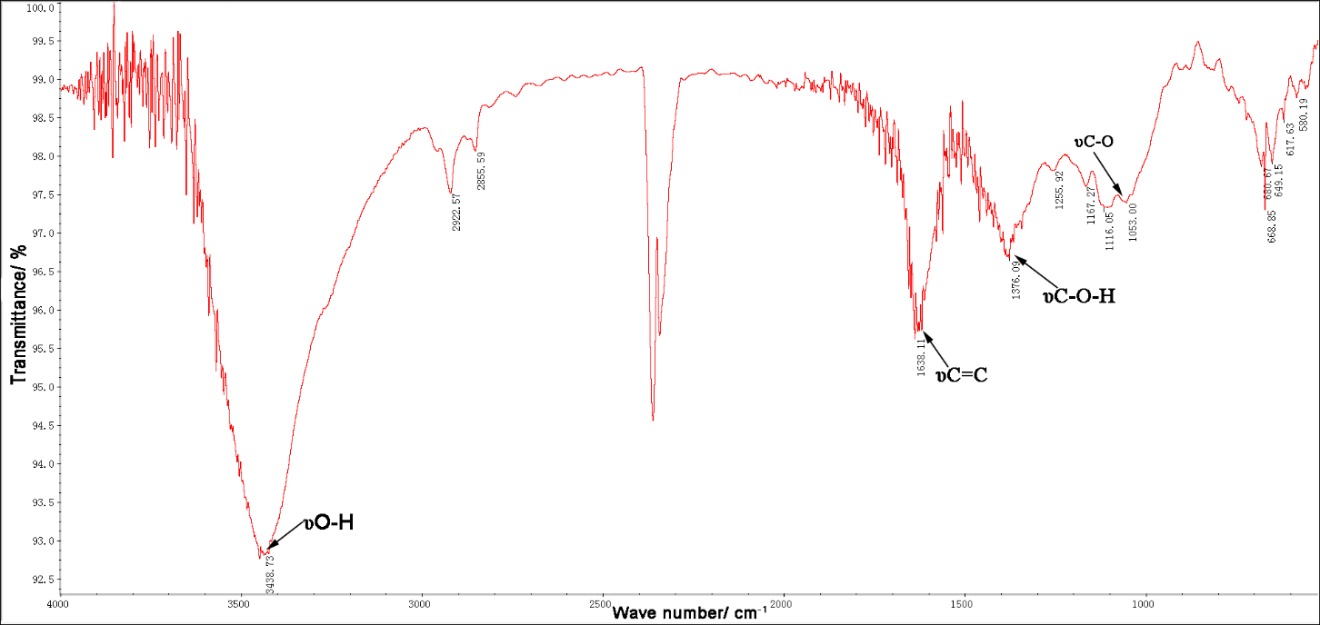
**Fig. S3** The FTIR spectra of fullerenols which stored at room temperature for one year
